# Supplementary material for: GLUT1 inhibition blocks growth of RB1-positive triple negative breast cancer
Source: Nat Commun. 2020 Aug 21;11:4205. doi: 10.1038/s41467-020-18020-8 (PMC7442809; doi:10.1038/s41467-020-18020-8)
Supplement: Supplementary file 1 — Supplementary Information [file 41467_2020_18020_MOESM1_ESM.pdf]

GLUT1 inhibition blocks growth of RB1-positive Triple Negative Breast Cancer

Wu et.al

Supplementary Table

Inhibitors	Target	Glut1 selectivity	Reference
STF31	GLUT1; NAMPT	n.d	[1,2]
WZB-117	GLUTs	inhibits glucose transport and cancer cell proliferation with IC_{50} of 10 µM	[3,4]
Fasentin	GLUT1/GLUT4; Fas-Sensitizer	preferentially inhibits GLUT4 (IC_{50}=68 µM) over GLUT1	[5]
BAY-876	GLUT1	oral bioavailable GLUT1 inhibitor with IC_{50} of 2 nM; displays >100-fold selectivity against GLUT2, GLUT3, and GLUT4	[6]

Supplementary Table 1: List of published inhibitors targeting glucose transporters 1. Chemical probes, their respective target names, selectivity for GLUT1 and the references.

Advantages and disadvantages of 3D patient relevant pre-clinical models			
	Organoids	Explants	Xenografts
Intact tissue architecture	✕	✓	✓
tumor heterogeneity	✓	✓	✓
Stromal-epithelial interactions	✕	✓	✓
Immune system	✕	limited	✕
Highthroughput drug screening	✓	✕	✕
Long term functional assays	✓	✕	✓
Quantitive measurements	✓	✓	limited

Supplementary Table 2: Table of advantages and disadvantages of 3D patient relevant pre-clinical models[7–12].

Target	Sequence
Rb1-Fw	TTGGATCACAGCGATACAAACTT
Rb1-Rv	AGCGCACGCCAATAAAGACAT
ATP5D-Fw	TCCCACGCAGGTGTTCTTC
ATP5D-Rv	GGAACCGCTGCTCACAAAGT
SLC25A22-Fw	GCCAGCCAAGCTCATCAATG
SLC25A22-Rv	GAGGCAGTCGGACATGCTC
AIFM1-Fw	CGTGACTATGTGTTAAGTTTCTCGC
AIFM1-Rv	GTAATGTGCGTGTGAAGAGACTG
ATP6V0B-Fw	ATCATCTTCTGTGAGGCTGTGGC
ATP6V0B-Rv	AGACTCCACAGAAGAGGTTAG ACAG
TOMM40-Fw	GAGTTTGAGGCCAGCACAAG

TOMM40-Rv	ACCCACGATCCAGTTGCTAT
PGC1α -Fw	TGAGAGGGCCAAGCAAAG
PGC1α -Rv	ATAAATCACACGGCGCTCTT
NDUFA9-Fw	GTCACGTTCTGCCATTACTGC
NDUFA9-Rv	GGTGGTTGACAACATATCGCC
TIMM17A-Fw	GGTGGGGCCTTTACGATGG
NDUFA9-Rv	GCCCTGGTTTTAATAGCTGTCA

Supplementary Table 3: List of primers for qRT-PCR.

Supplementary Figures:

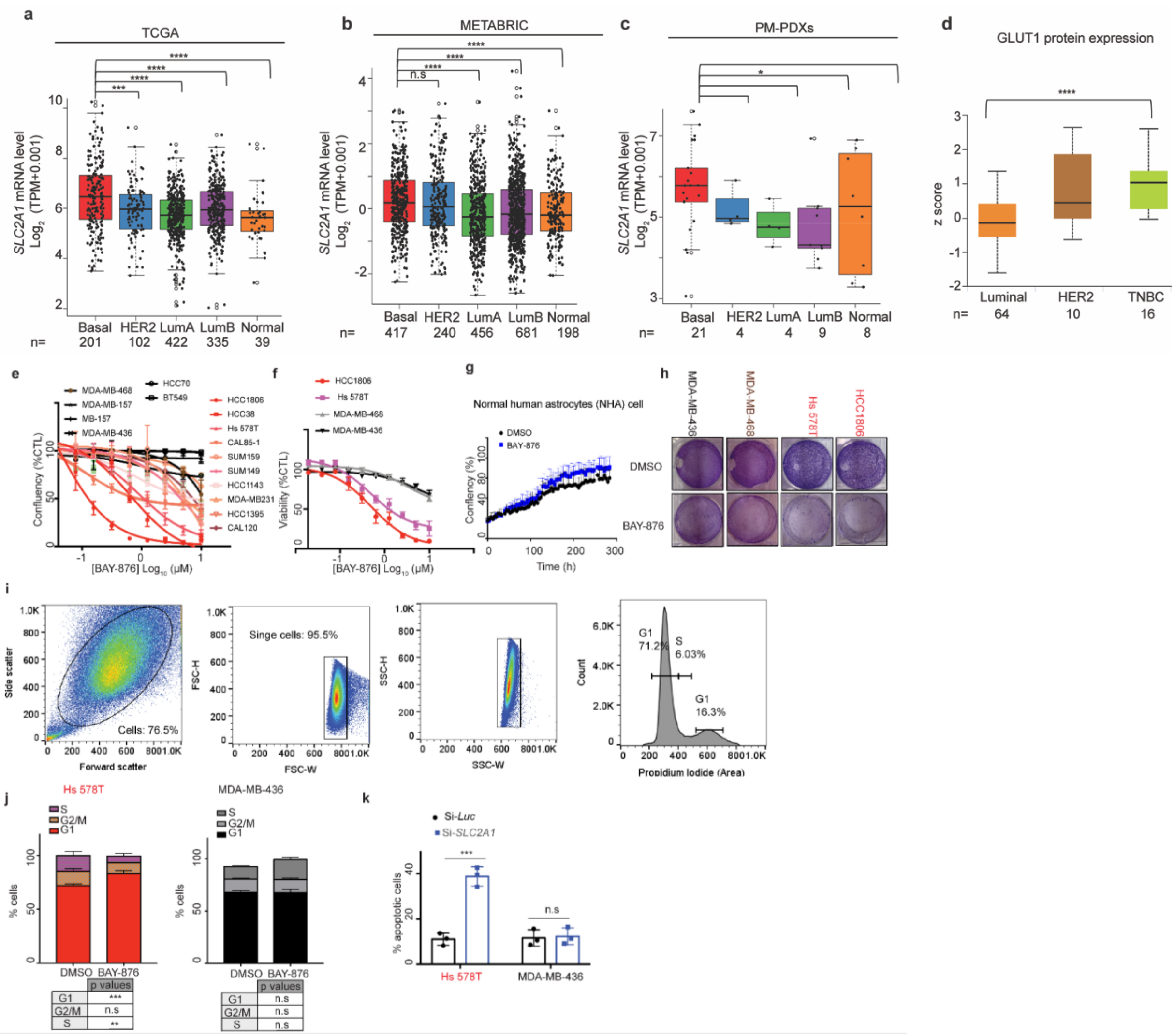

Supplementary Figure1: GLUT1 inhibition suppresses the growth of a subset of TNBC lines. **(a)** *SLC2A1* gene expression in the **(a)** TCGA Breast datasets, **(b)** METABRIC Breast cancer datasets, and **(c)** Princess Margret Hospital PDXs datasets (PM-PDXs). According to PAM50 classification, the cohorts were designated as basal, HER2, LumA, LumB and normal. Gene expression is reported as log_2(TPM+0.001). The number of patients (n) per group is indicated. Wilcoxon rank sum test. *p<0.05; **p<0.01; ***p<0.001; ****p<0.0001. **(d)** GLUT1 protein expression in Clinical Proteomic Tumor Analysis Consortium (CPTAC) Confirmatory/Discovery dataset. Z-values represent standard deviations from the median across samples for the given cancer type. Log2 Spectral count ratio values from CPTAC were first normalized within each sample profile, then normalized across samples. ****p<0.0001. **(e)** Growth curves of 17 breast cancer lines with indicated concentration of BAY-876 treatment for 5 days. **(f)** Cell viability assays five days after administration of DMSO control or the indicated concentrations doses of BAY-876 across four TNBC cell lines. n=4; mean ± s.d.**(g)**.Growth curves of normal human astrocytes (NHA) cells treated with DMSO or 5 μM BAY-876. **(h)** Long-term colony formation assays of cell lines deemed sensitive (HCC1806 and Hs578T) and insensitive lines (MDA-MB436 and MDA-MB468) in the short-term viability assays. **(i)** Representative plots for gating strategy of cell cycle analysis. **(j)** Flow cytometry cell cycle analysis of indicated cells with GLUT1 knockdown. n=3; mean ±

s.d. Two-way ANOVA. **p<0.01; ***p<0.001; n.s means not significant. **(k)** Apoptotic cell counts of GLUT1 knockdown cells or siRNA luciferase control cells by caspase 3/7 staining. n=3; mean ± s.d. Two-way ANOVA. ***p<0.001; n.s means not significant. Source data are provided as a Source Data file.

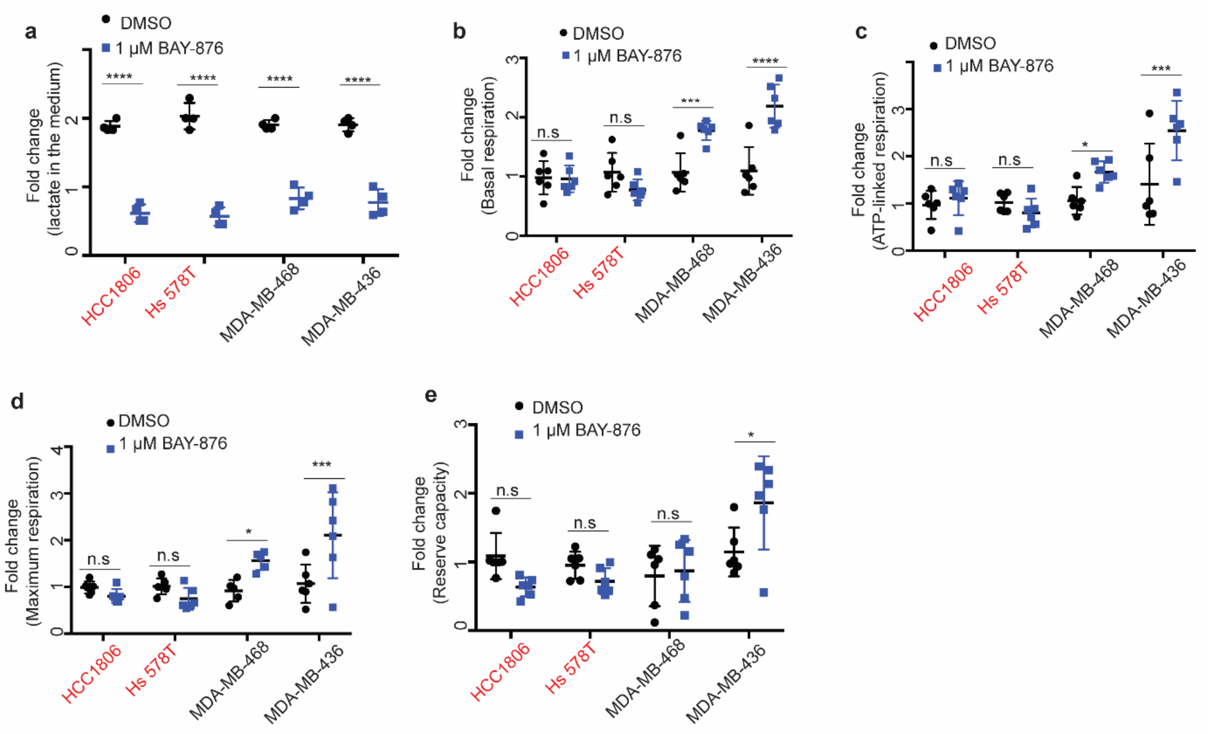

Supplementary Figure2: Cell metabolism levels correlate with response to GLUT1 inhibition. **(a)** Lactate uptake analysis using Bioprofile Flex analyzer (Nova Biomedical) were performed in cells following BAY-876 treatment for 5 days. Mean ± s.d; n = 3. A trace of **(b)** basal respiration; **(c)** ATP-linked respiration; **(d)** maximum respiration and **(e)** reserve capacity values from a mitochondrial stress test of cells with or without BAY-876 treatment for 5 days. P values computed using a two-way ANOVA. *p<0.01; *p<0.5; ***p<0.001; ****p<0.0001; n.s denotes not significant.

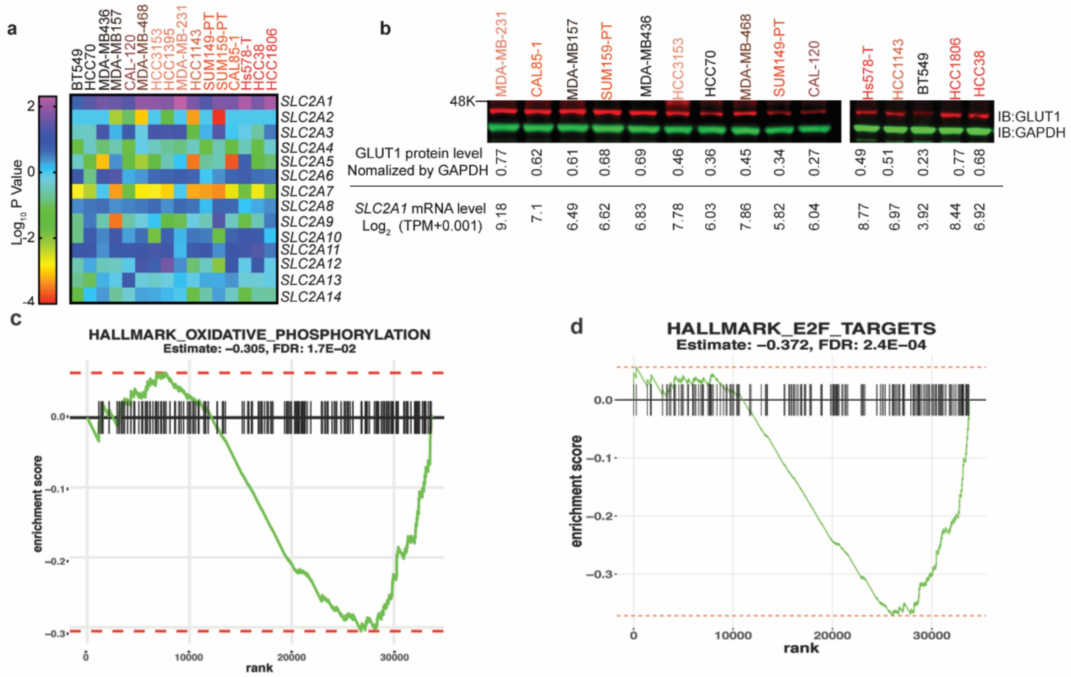

e

Basal Hallmarks Correlated with Hallmark OXPHOS

	sort(CorWithPath_HALLMARK[, 1], decreasing = T)
HALLMARK_PEROXISOME	0.460675336190335
HALLMARK_FATTY_ACID_METABOLISM	0.428638983301315
HALLMARK_DNA_REPAIR	0.404737697650362
HALLMARK_MYC_TARGETS_V2	0.384078616816906
HALLMARK_BILE_ACID_METABOLISM	0.378307965124871
HALLMARK_MYC_TARGETS_V1	0.344931284173193
HALLMARK_SPERMATOGENESIS	0.344375646519876
HALLMARK_MTORC1_SIGNALING	0.324480567459731
HALLMARK_ADIPOGENESIS	0.319843357470075
HALLMARK_HEME_METABOLISM	0.27556672085119
HALLMARK_GLYCOLYSIS	0.252676222846165
HALLMARK_REACTIVE_OXIGEN_SPECIES_PATHWAY	0.250112309738437
HALLMARK_UNFOLDED_PROTEIN_RESPONSE	0.24350229052756
HALLMARK_E2F_TARGETS	0.233970740357618
HALLMARK_CHOLESTEROL_HOMEOSTASIS	0.214258903502291
HALLMARK_PANCREAS_BETA_CELLS	0.212948130633959
HALLMARK_XENOBIOTIC_METABOLISM	0.206559775380523
HALLMARK_UV_RESPONSE_UP	0.185430766957293
HALLMARK_G2M_CHECKPOINT	0.175616964681543
HALLMARK_ESTROGEN_RESPONSE_LATE	0.167629673415103
HALLMARK_ESTROGEN_RESPONSE_EARLY	0.162795921383183

Supplementary Figure3: RB1-E2F pathway significantly correlates with BAY-876 sensitivity. **(a)** Correlation heatmap of expression level of 14 glucose transporters and BAY-876 sensitivity. Colour grading of cell lines corresponds to non-responders (black) and non-responders (red) according to the IC_{50} of BAY-876. **(b)** Representative western blot showing the variable GLUT1 expression levels in a panel of 15 TNBC lines. GAPDH was used as a loading control for normalization. Band intensities were quantified using Image J software and the corresponding values were labelled. *SLC2A1* mRNA expression level was labelled as well based on RNA-sequencing data. **(c)** OXPHOS as a significant enriched pathway in non-responders compared to responders based on GSEA on RNA-sequencing data generated. **(d)** E2F Targets as a significant enriched pathway in non-responders compared to responders based on GSEA on RNA-sequencing data generated. **(e)** Top correlated pathways correlated with OXPHOS in TCGA dataset cohort. Source data are provided as a Source Data file.

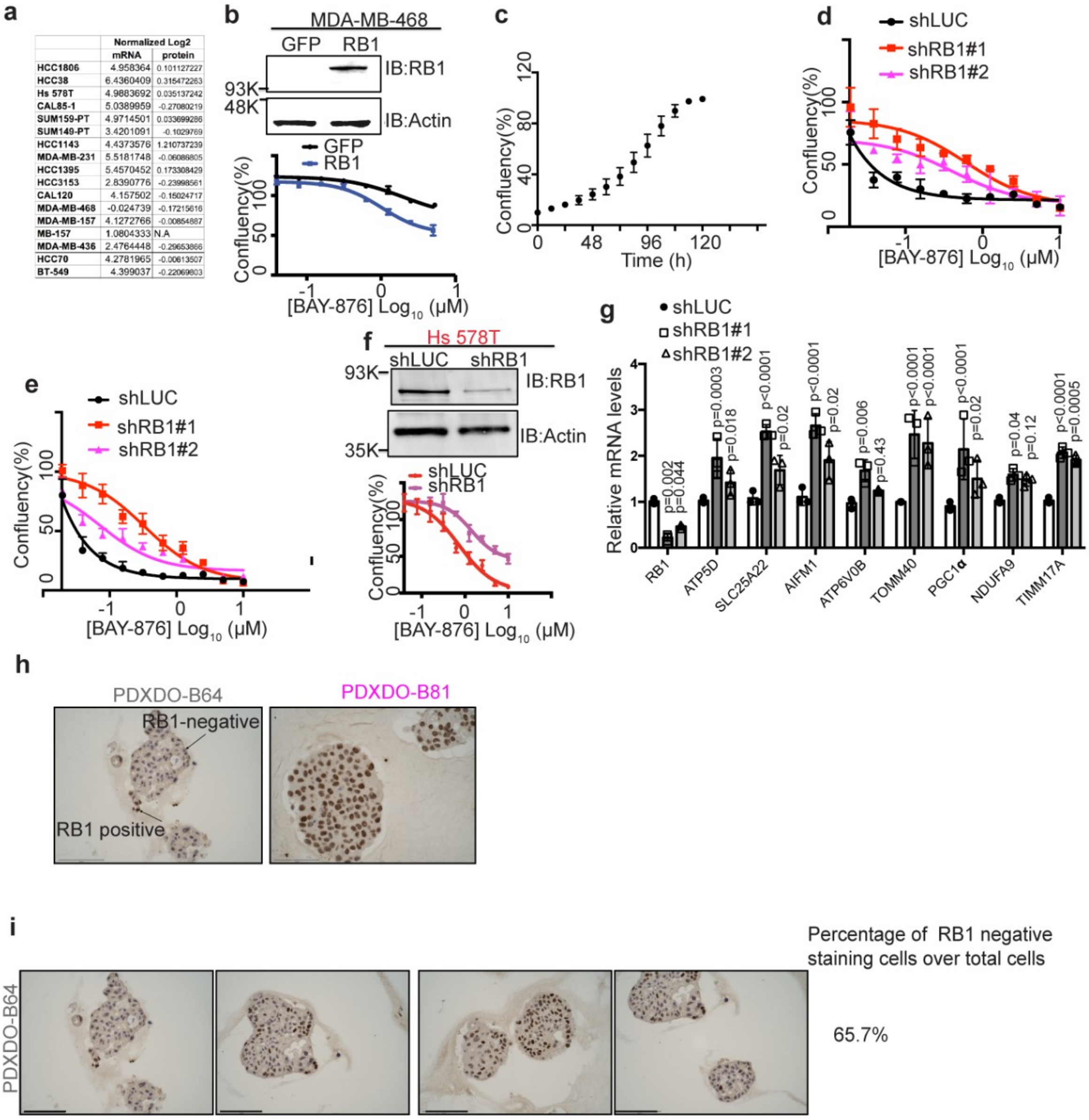

	Normalized Log2	
	mRNA	protein
HCC1806	4.958364	0.101127227
HCC38	6.4360409	0.315472263
Hs 578T	4.9883692	0.035137242
CAL85-1	5.0389959	-0.27080219
SUM159-PT	4.9714501	0.033699286
SUM149-PT	3.4201091	-0.1029769
HCC1143	4.4373576	1.210737239
MDA-MB-231	5.5181748	-0.06086805
HCC1395	5.4570452	0.173308429
HCC3153	2.8390776	-0.23998561
CAL120	4.157502	-0.15024717
MDA-MB-468	-0.024739	-0.17215616
MDA-MB-157	4.1272766	-0.00854887
MB-157	1.0804333	N.A
MDA-MB-436	2.4764448	-0.29653866
HCC70	4.2781965	-0.00613507
BT-549	4.399037	-0.22069803

Supplementary Figure4: RB1 levels dictates BAY876 sensitivity in TNBC. **(a)** RB1 mRNA and protein level for indicated cell lines. **(b)** Upper panel: Representative western blot showing MDA-MB468 cells expressing RB1 or GFP control proteins. β-actin was used as a loading control. Bottom panel: Growth curves of MDA-MB-468 cells expressing RB1 or GFP control in the presence of indicated concentrations BAY876 treatment for 5 days. mean ± s.d; n = 4. **(c)** Growth curves of HCC1806 cells with DMSO for indicated time culture. mean ± s.d; n = 4. **(d)** Growth curves of HCC1806 cells with control knockdown or RB1 knockdown in the presence of indicated concentrations BAY-876 treatment for 48h. mean ± s.d; n = 4. **(e)** Growth curves of HCC1806 cells with control knockdown or RB1 knockdown in the presence of indicated concentrations BAY-876 treatment for 72h. mean ± s.d; n = 4. **(f)** Upper panel: Representative western blot showing Hs 578 T cells transfected with shRNA targeting RB1 or control luciferase. β-actin as a loading control. Bottom panel: Growth curves of Hs 578T cells with control knockdown or RB1 knockdown in the presence of indicated concentrations BAY-876 treatment for 5 days. mean ± s.d; n = 4. **(g)** Relative mRNA levels of mitochondrial genes in HCC1806-shRB1 cells compared to control measured by qRT-PCR. Mean ± s.e.m., n = 3, two-tailed t-test. **(h)** Representative images

of RB1 IHC staining for PDXDOs. Arrows indicate different cells showing distinct staining intensity. Scale bars represent 100 µm. **(i)** Images of RB1 IHC staining for RB1 negative PDXDOB 64. Scale bars represent 100 µm. Percentage of RB1 negative staining cells were calculated. Source data are provided as a Source Data file.

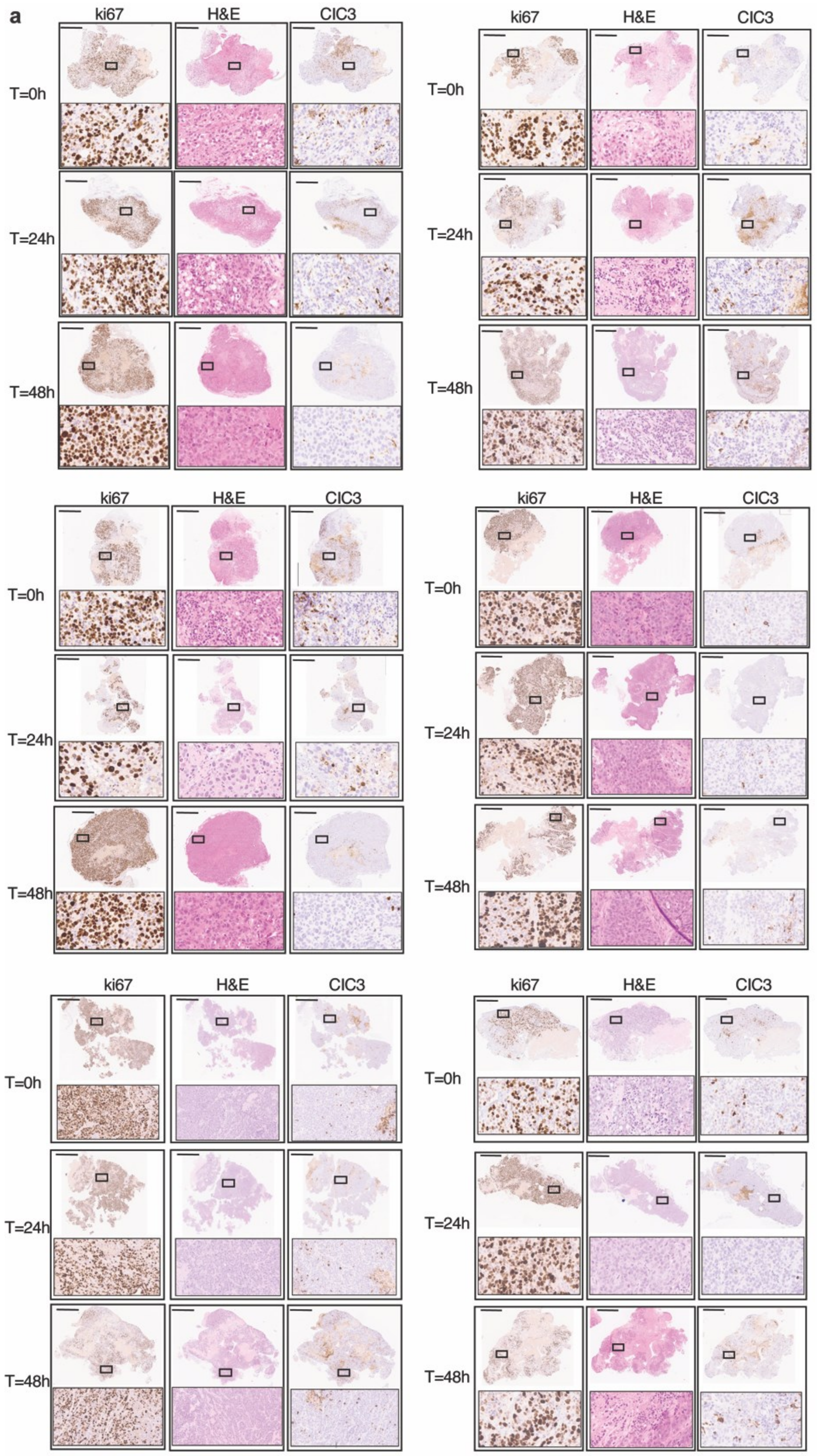

Supplementary Figure5: Viability of explants during *ex vivo* culture time range. **(a)** Viability of explants during *ex vivo* culture time range assessed by proliferative index ki67 staining, H&E

staining and cleaved caspase 3 (ClC3) staining. Scale bars represent 500 µm. Indicated area is zoomed in 5X at the bottom.

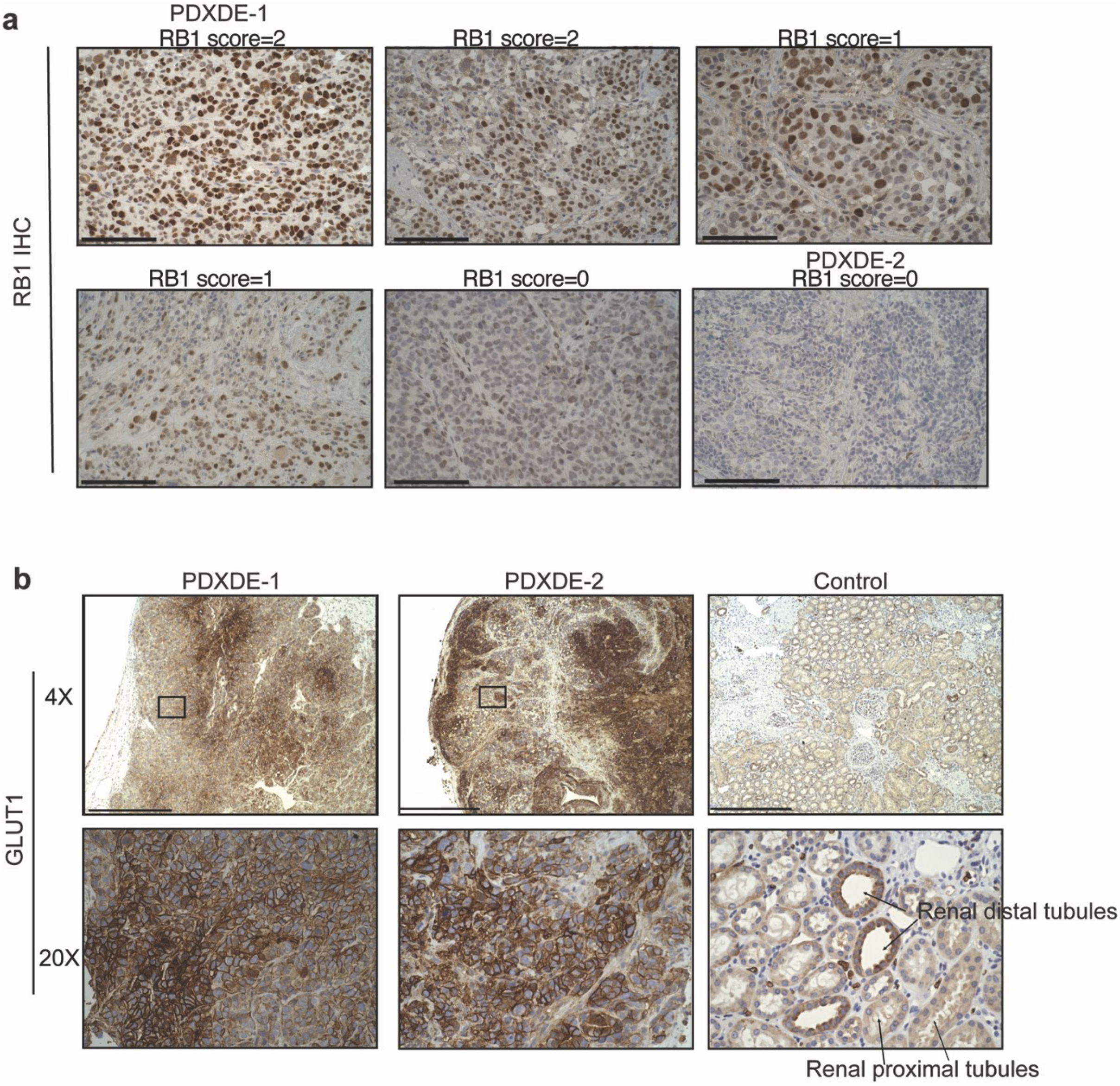

Supplementary Figure6: On-target effect of BAY-876 in PDXDEs. **(a)** Representative immunohistochemistry staining images of six PDXDEs from different TNBC patients. Scale bars represent 100 µm. RB1 IHC scores as indicated. **(b)** Representative GLUT1 IHC staining images of PDXDE-1, PDXDE-2. GLUT1 staining of human kidney tissue as negative control and positive control. Renal proximal tubules cells lacking GLUT1 expression as negative control, while renal distal tubules cells as positive control [13]. Scale bars represent 500 µm. Indicated area is zoomed in 5X at the bottom.

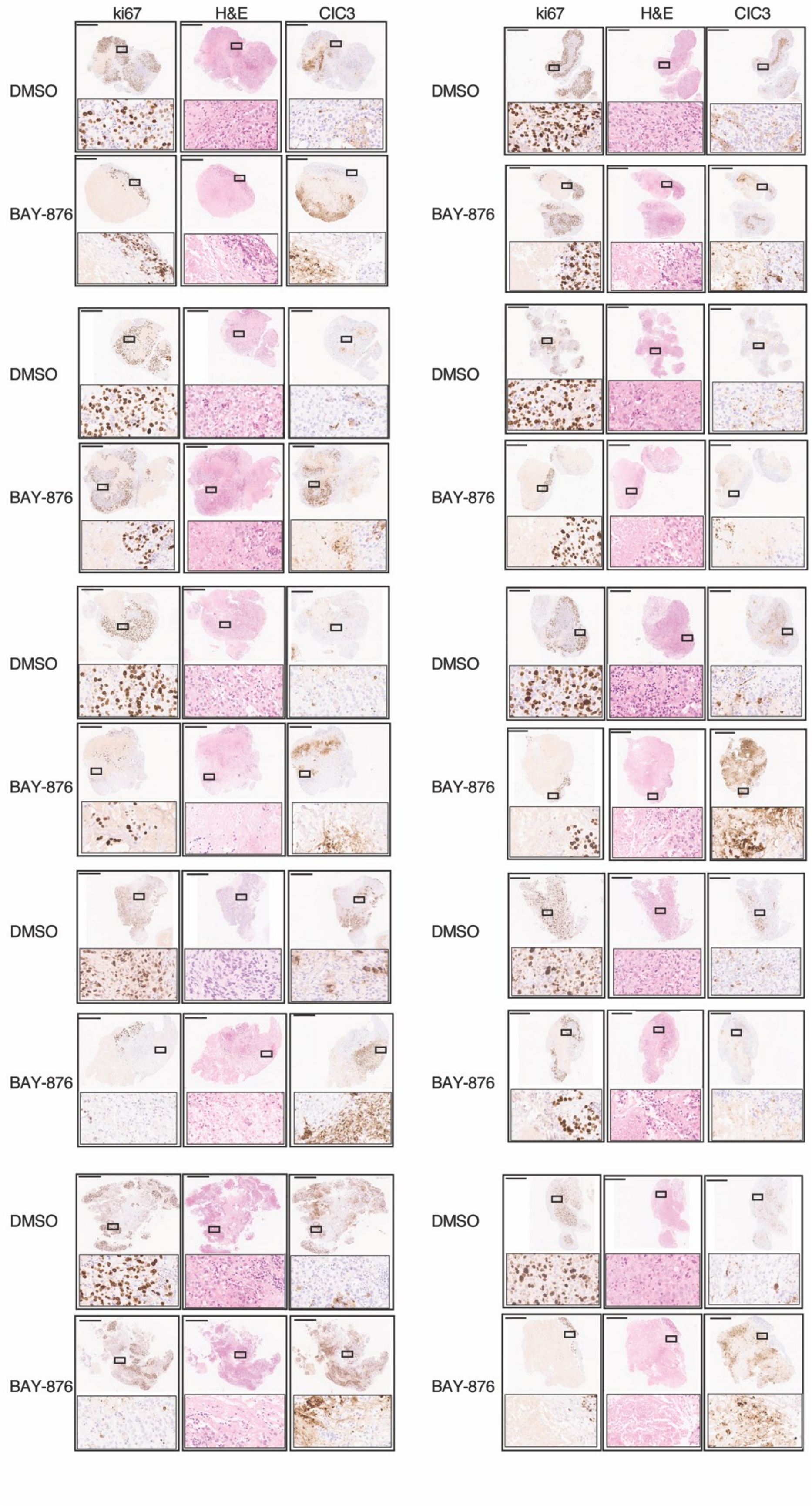

Supplementary Figure7. IHC staining images of PDXDE-1. Scale bar represents 500 µm. Indicated area is zoomed in 5X at the bottom.

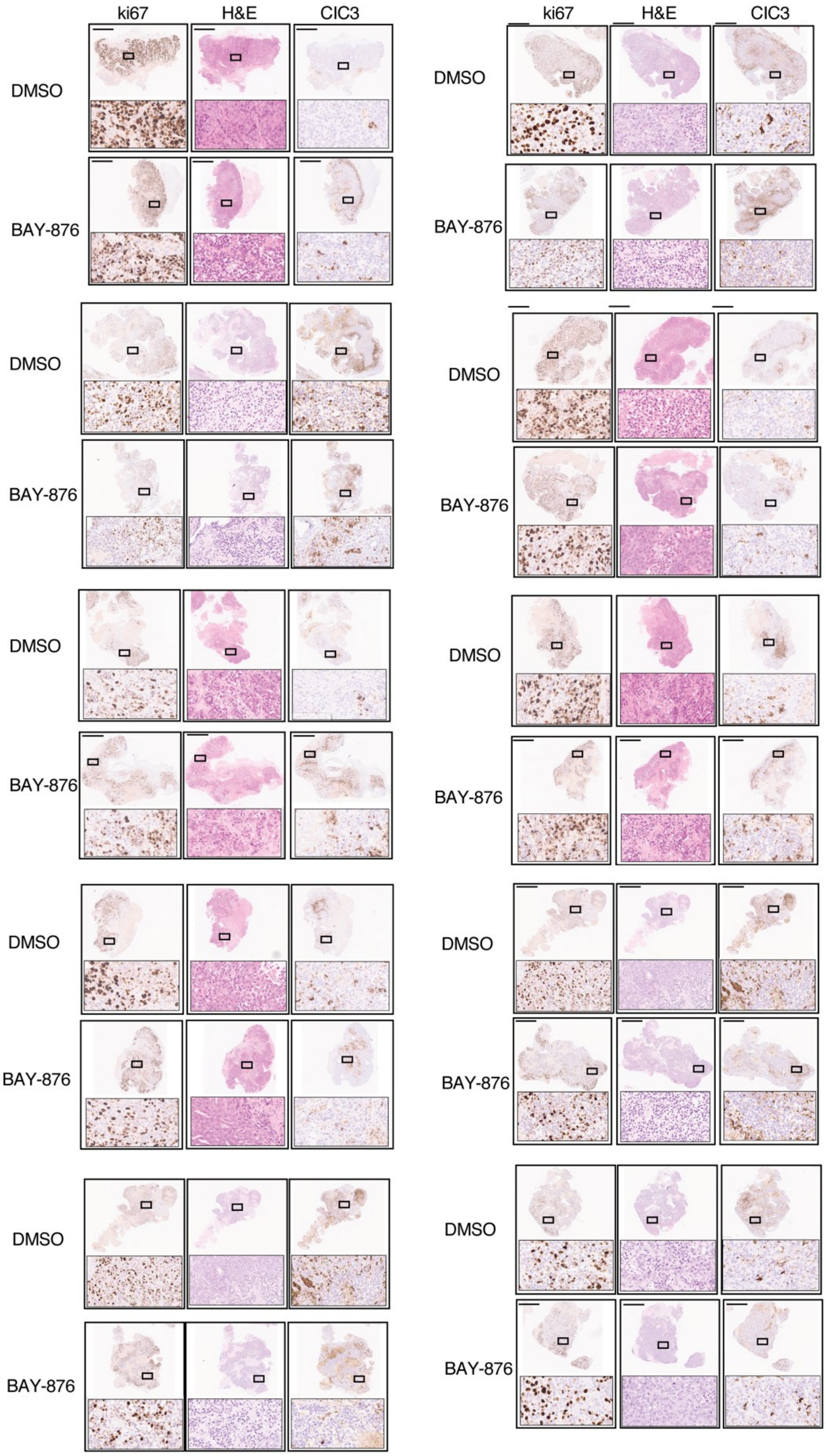

Supplementary Figure8. IHC staining images of PDXDE-2. Scale bar represents 500 μm. Indicated area is zoomed in 5X at the bottom.

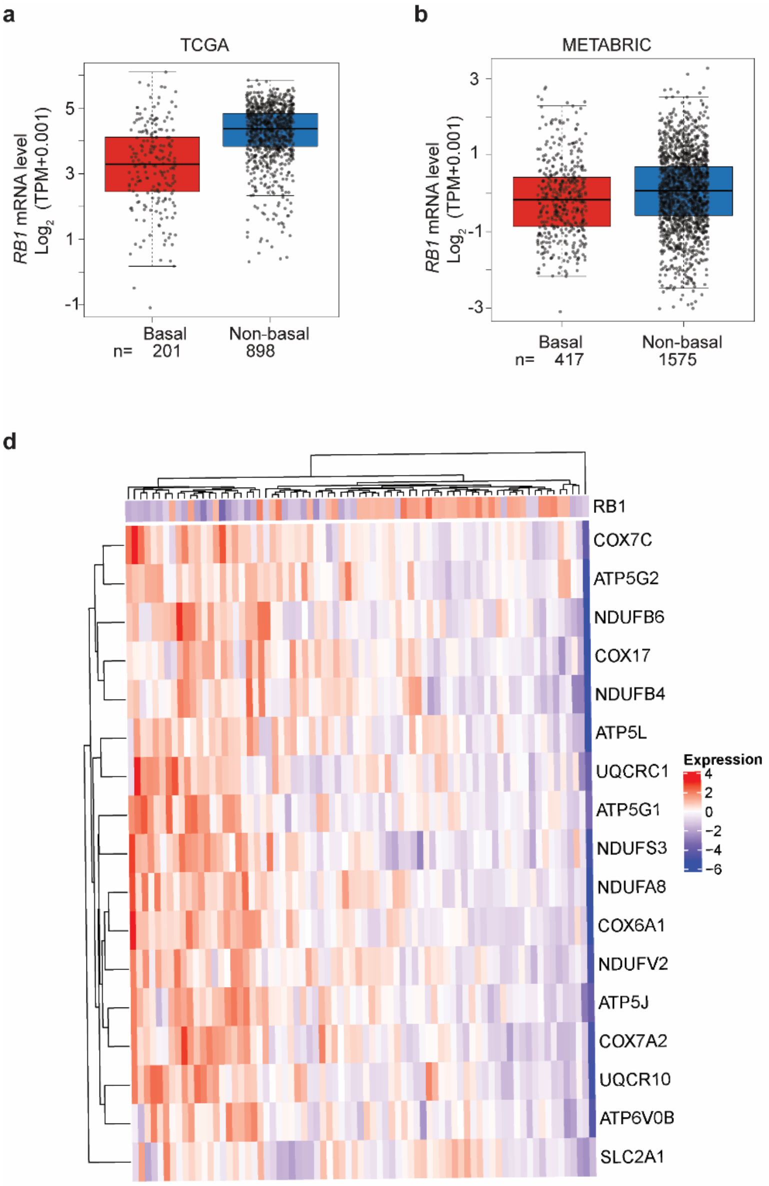

Supplementary Figure9: *RB1* gene expression in the **(a)** TCGA Breast cancer datasets, **(b)** METABRIC Breast cancer datasets. Gene expression is reported as $\log_2$(TPM+0.001). The number of patients (n) per group is indicated. *P* values were determined using a Wilcoxon rank sum test. * p<0.05; ****p<0.0001. RBN indicates replicates-based normalization. **(c)** RB1 protein level in TCGA Breast cancer datasets. The number of patients (n) per group is indicated. **(d)** Heatmap showing RB1 expression is negatively correlated with OXPHOS genes expression for each patient with TNBC tumors (TCGA provisional dataset).

Supplementary References

1. Chan, D. A. *et al.* Targeting GLUT1 and the Warburg effect in renal cell carcinoma by chemical synthetic lethality. *Sci. Transl. Med.* **3**, 94ra70 (2011).

2. Adams, D. J. *et al.* NAMPT is the cellular target of STF-31-like small-molecule probes. *ACS Chem. Biol.* **9**, 2247–2254 (2014).

3. Liu, Y. *et al.* A small-molecule inhibitor of glucose transporter 1 downregulates glycolysis, induces cell-cycle arrest, and inhibits cancer cell growth in vitro and in vivo. *Mol. Cancer Ther.* **11**, 1672–1682 (2012).
4. Ojelabi, O. A., Lloyd, K. P., Simon, A. H. & De Zutter, J. K. WZB117 inhibits GLUT1-mediated sugar transport by binding reversibly at the exofacial sugar binding site. *Journal of Biological* (2016).
5. Ocaña, M. *et al.* Fasentin, a glucose uptake inhibitor, is also able to inhibit angiogenesis. (2017).
6. Siebeneicher, H. *et al.* Identification and Optimization of the First Highly Selective GLUT1 Inhibitor BAY-876. *ChemMedChem* **11**, 2261–2271 (2016).
7. Lee, J.-K. *et al.* Pharmacogenomic landscape of patient-derived tumor cells informs precision oncology therapy. *Nat. Genet.* **50**, 1399–1411 (2018).
8. Collins, A. *et al.* Patient-derived explants, xenografts and organoids: 3-dimensional patient-relevant pre-clinical models in endometrial cancer. *Gynecol. Oncol.* **156**, 251–259 (2020).
9. Centenera, M. M. *et al.* A patient-derived explant (PDE) model of hormone-dependent cancer. *Mol. Oncol.* **12**, 1608–1622 (2018).
10. Bruna, A. *et al.* A Biobank of Breast Cancer Explants with Preserved Intra-tumor Heterogeneity to Screen Anticancer Compounds. *Cell* **167**, 260–274.e22 (2016).
11. Powley, I. R. *et al.* Patient-derived explants (PDEs) as a powerful preclinical platform for anti-cancer drug and biomarker discovery. *Br. J. Cancer* (2020) doi:10.1038/s41416-019-0672-6.
12. Louandre, C. *et al.* Personalization of the medical treatment of solid tumours using patient-derived tumour explants (Review). *International Journal of Oncology* vol. 48 895–899 (2016).
13. Ozcan, A., Shen, S. S., Zhai, Q. J. & Truong, L. D. Expression of GLUT1 in primary renal tumors: morphologic and biologic implications. *Am. J. Clin. Pathol.* **128**, 245–254 (2007).
