# Supplementary material for: GLUT1 inhibition blocks growth of RB1-positive triple negative breast cancer
Source: Nat Commun. 2020 Aug 21;11:4205. doi: 10.1038/s41467-020-18020-8 (PMC7442809; doi:10.1038/s41467-020-18020-8)
Supplement: Supplementary file 2 — Reporting summary [file 41467_2020_18020_MOESM2_ESM.pdf]

nature research

Corresponding author(s): Cheryl Arrowsmith, Mathieu Lupien

Last updated by author(s): Jul 8, 2020

Reporting Summary

Nature Research wishes to improve the reproducibility of the work that we publish. This form provides structure for consistency and transparency in reporting. For further information on Nature Research policies, see Authors & Referees and the Editorial Policy Checklist .

Statistics

For all statistical analyses, confirm that the following items are present in the figure legend, table legend, main text, or Methods section.

n/a	Confirmed	
☐	☒	The exact sample size (*n*) for each experimental group/condition, given as a discrete number and unit of measurement
☐	☒	A statement on whether measurements were taken from distinct samples or whether the same sample was measured repeatedly
☐	☒	The statistical test(s) used AND whether they are one- or two-sided<br>*Only common tests should be described solely by name; describe more complex techniques in the Methods section.*
☐	☒	A description of all covariates tested
☐	☒	A description of any assumptions or corrections, such as tests of normality and adjustment for multiple comparisons
☐	☒	A full description of the statistical parameters including central tendency (e.g. means) or other basic estimates (e.g. regression coefficient) AND variation (e.g. standard deviation) or associated estimates of uncertainty (e.g. confidence intervals)
☒	☐	For null hypothesis testing, the test statistic (e.g. *F*, *t*, *r*) with confidence intervals, effect sizes, degrees of freedom and *P* value noted<br>*Give P values as exact values whenever suitable.*
☒	☐	For Bayesian analysis, information on the choice of priors and Markov chain Monte Carlo settings
☒	☐	For hierarchical and complex designs, identification of the appropriate level for tests and full reporting of outcomes
☐	☒	Estimates of effect sizes (e.g. Cohen's *d*, Pearson's *r*), indicating how they were calculated

Our web collection on statistics for biologists contains articles on many of the points above.

Software and code

Policy information about availability of computer code

Data collection	Incucyte Zoom 2016A<br>Odyssey scanner (LiCor)<br>BD FACScan flow cytometer<br>Seahorse XF96 extracellular flux Bioanalyzer<br>Bioprofile Flex analyzer (Nova biomedical)<br>BenchMark XT-an automated slide stainer<br>Aperio ScanScope AT2 at 20x magnification
Data analysis	Incucyte ZOOM 2016A<br>Prism Version 7.0c<br>R (V.3.5.1)<br>Bioconductor (v.3.7)<br>FlowJo software (version 9.3.1)<br>ExcelMacro Report Generator Version 3.0.3<br>IncucyteDRC R package<br>Definiens TissueStudio 4.3 software (Definiens Inc., Munich Germany)

For manuscripts utilizing custom algorithms or software that are central to the research but not yet described in published literature, software must be made available to editors/reviewers. We strongly encourage code deposition in a community repository (e.g. GitHub). See the Nature Research guidelines for submitting code & software for further information.

Data

Policy information about availability of data

All manuscripts must include a data availability statement. This statement should provide the following information, where applicable:

- Accession codes, unique identifiers, or web links for publicly available datasets
- A list of figures that have associated raw data
- A description of any restrictions on data availability

The data supporting the findings of this study are available within the paper and its supplementary information files and are available from the corresponding authors. Code to reproduce the bioinformatics analyses and their related data is available at https://github.com/bhklab/TNBC_BAY-876.

Field-specific reporting

Please select the one below that is the best fit for your research. If you are not sure, read the appropriate sections before making your selection.

[x] Life sciences [ ] Behavioural & social sciences [ ] Ecological, evolutionary & environmental sciences

For a reference copy of the document with all sections, see nature.com/documents/nr-reporting-summary-flat.pdf

Life sciences study design

All studies must disclose on these points even when the disclosure is negative.

Sample size	Sample sizes of 3 biological replicates and two or three individual experiments are sufficient to determine significant differences for changes in the analyzed biological products.
Data exclusions	No data has been excluded from this study
Replication	All findings using the inhibitors to alter growth of cell lines, organoids and explants were reproducible over several experiment.
Randomization	Randomization was not relevant to this study since no mice work is included in this work.
Blinding	Blinding was not relevant to this study as all biological samples were equally analyzed.

Reporting for specific materials, systems and methods

We require information from authors about some types of materials, experimental systems and methods used in many studies. Here, indicate whether each material, system or method listed is relevant to your study. If you are not sure if a list item applies to your research, read the appropriate section before selecting a response.

Materials & experimental systems

n/a	Involved in the study	
[ ]	[x]	Antibodies
[ ]	[x]	Eukaryotic cell lines
[x]	[ ]	Palaeontology
[ ]	[x]	Animals and other organisms
[x]	[ ]	Human research participants
[x]	[ ]	Clinical data

Methods

n/a	Involved in the study	
[x]	[ ]	ChIP-seq
[ ]	[x]	Flow cytometry
[x]	[ ]	MRI-based neuroimaging

Antibodies

Antibodies used

anti-GLUT1 rabbit monoclonal (EPR3915) (ab115730) (1:1000)
anti-RB1 rabbit monoclonal (EPR) (ab181616) (1:2000)
anti-beta actin (ab16039) (1:5000)
goat-anti rabbit (IR800 conjugated, LiCor no. 926-32211)
donkey anti-mouse (IR 680, LiCor no. 926-68072) antibodies (1:5000).
anti-Ki67 (Dako M7240, clone MIB1) (1:100)
cleaved caspase 3 (cell signalling #9661) (1:500)
Glut1 (Roche #06419178001) (1:1000)
RB (BD #554136) (1:1,600)

Validation	see websites of the manufacturers

Eukaryotic cell lines

Policy information about cell lines

Cell line source(s)	ATCC, Lonza
Authentication	none were authenticated
Mycoplasma contamination	All cell lines tested negative for mycoplasma
Commonly misidentified lines (See ICLAC register)	No

Animals and other organisms

Policy information about studies involving animals; ARRIVE guidelines recommended for reporting animal research

Laboratory animals	SCID mice
Wild animals	This study did not involve wild animals.
Field-collected samples	This study did not involve field-collected samples
Ethics oversight	All animal experiments were reviewed and approved by the Animal Care Committee at the University Health Network in Toronto.

Note that full information on the approval of the study protocol must also be provided in the manuscript.

Flow Cytometry

Plots

Confirm that:

- [x] The axis labels state the marker and fluorochrome used (e.g. CD4-FITC).
- [x] The axis scales are clearly visible. Include numbers along axes only for bottom left plot of group (a 'group' is an analysis of identical markers).
- [x] All plots are contour plots with outliers or pseudocolor plots.
- [x] A numerical value for number of cells or percentage (with statistics) is provided.

Methodology

Sample preparation	Exponentially growing cells in six-well plates were treated with 3 µM BAY-876 or DMSO for 24h before cell cycle analysis using allophycocyanin (APC) BrdU Flow kit (BD Pharmingen). Briefly, cells were incubated with 10 µM BrdU for 6 h before fixation, permeabilization, and staining with APClabeled anti-BrdU antibody and 7-aminoactinomycin D (7- AAD) according to the manufacturer's instructions. Cells were then analyzed using a BD FACScan flow cytometer and the percentage of live cells in each cell cycle stage was determined using FlowJo software (version 9.3.1).
Instrument	BD FACScan flow cytometer
Software	FlowJo software (version 9.3.1)
Cell population abundance	Cell population abundance has been indicated in the table in the main figure.
Gating strategy	Gating strategy are shown in the supplementary figures.

- [x] Tick this box to confirm that a figure exemplifying the gating strategy is provided in the Supplementary Information.
